# No Laughing Matter: How Humor Styles Relate to Feelings of Loneliness and Not Mattering

**DOI:** 10.3390/bs10110165

**Published:** 2020-10-27

**Authors:** Kristi Baerg MacDonald, Anjali Kumar, Julie Aitken Schermer

**Affiliations:** 1 Psychology Department, The University of Western Ontario, London, ON N6A 5C2, Canada; kmacd252@uwo.ca (K.B.M.); angie.kumar@hotmail.com (A.K.); 2 Management and Organizational Studies, Faculty of Social Science, The University of Western Ontario, London, ON N6A 5C2, Canada

**Keywords:** adult, humor, humor styles, loneliness, mattering, anti-mattering

## Abstract

Loneliness and feeling that one does not matter are closely linked, but further investigation is needed to determine differentiating features. The relationship between not mattering to others (anti-mattering) and loneliness was explored by assessing how the two constructs correlated with an interpersonal dimension, specifically four humor styles (affiliative, self-enhancing, self-defeating, and aggressive). One hundred and fifty-eight women and 96 men completed a three-item loneliness scale, a new measure of anti-mattering, and a humor styles questionnaire. Confirmatory factor analysis results indicated that the new anti-mattering measure is a unidimensional scale. Loneliness and anti-mattering were strongly correlated, and each correlated in the same direction with approximately the same magnitude as the four humor styles. The discussion concludes that anti-mattering and loneliness are strongly linked, a finding which may be important in psychological treatment. Humor styles also play a role in psychological well-being and present a unique pathway to mental health.

## 1. Introduction

The feeling that one matters was first examined by Rosenberg and McCullough [1] and has fueled research on how “mattering” develops and how it impacts people, present and future. Mattering is defined as having others who depend on you, are interested in you, and care about what happens to you [1]. The converse to this occurs when people feel that they do not matter to those in their lives. Mattering has been shown to be negatively associated with mental health problems, particularly with loneliness [2]. Loneliness and mattering have been found to have a robust correlation (*r* = −0.65), suggesting that the two terms might be describing one concept [2]. If mattering is found to correlate similarly in both direction and magnitude with the correlations between loneliness and a third interpersonal dimension, these correlations may further indicate that loneliness and mattering are synonymous or fall under a common construct. The present study addresses whether loneliness differs from not mattering (anti-mattering) based on correlations with humor styles.

### 1.1. Humor Styles

Humor styles are unique, but often over-looked, variables that also have considerable associations with measures of mental health. While on the surface, people associate humor with happiness and levity, some humor styles point to loneliness and depression. In the development of the Humor Styles Questionnaire (HSQ), Martin et al. [3] provided a model in the form of a 2 × 2 conceptualization of humor functions. The humor styles model distinguishes between humor that affects the self and humor that affects others. The other dimension is humor that is enhancing and humor that is disparaging. These distinctions result in four different styles. The enhancing styles include affiliative and self-enhancing and are related to overall happiness and psychological hardiness [4]. They are also both significantly related to social support, which acts as a mediator from humor styles to life satisfaction [5]. Affiliative humor style is focused on others and refers to the tendency to use humor in a positive manner and to increase group cohesiveness and enhance one’s relationships with others (example item, “I enjoy making people laugh”). When people use humor to cheer themselves, they are engaging in self-enhancing humor (example item, “Even when I’m by myself, I’m often amused by the absurdities of life”). In contrast, the disparaging humor styles include aggressive and self-defeating humor. Aggressive humor styles involve belittling others (example item, “If someone makes a mistake, I will often tease them about it”), and while it can have the intent of developing group identity, aggressive humor can also harm personal relationships [3]. Self-defeating humor styles involve using the self as the target of ridicule, which may be used to ingratiate oneself with others (example item, “I will often get carried away in putting myself down if it makes my family or friends laugh”). Of interest, Schermer et al. [6] reported a strong genetic correlation between the self-defeating humor style and loneliness, suggesting that the two constructs may have a common genetic factor.

### 1.2. Loneliness

Loneliness comprises psychological and interpersonal factors, much like mattering, lending itself well to comparisons with humor styles. Hampes [7] reported finding that affiliative and self-enhancing humor were negatively associated with loneliness, self-defeating humor was positively associated with loneliness, and aggressive humor was not significantly associated with loneliness. This pattern of results was later replicated by Ceçen [8], Fitts et al. [9], and Schermer et al. [6]. These results suggest that humor styles and loneliness have meaningful correlations, but less is known about how mattering is related to humor styles.

While loneliness is an experience across the lifespan, research has generally found a U-shaped relationship; that is, greater loneliness in adults under age 25 and over age 65 [10,11]. Young adulthood is a time of significant change, where people are often leaving public school, moving away from family, starting new employment, or experiencing friends move away. Rokach [12] found that compared to other ages, adults in their 20s experience significantly more distress from loneliness than other age groups.

### 1.3. Mattering

The field of positive psychology suggests that individuals will succeed in their lives to the degree that they believe they matter to others. Mattering is also associated with many aspects of positive affect. France and Finney [13] found that four elements of mattering—being the focus of another person’s attention (awareness), being the focus of another person’s concern (importance), feeling needed by people in one’s life (reliance), and feeling like a part of another person (extension)—were significantly correlated with purpose in life, self-esteem, and positive interpersonal relationships. Further, mattering has been found to be strongly associated with mastery, growth, and autonomy [14].

In terms of hedonic wellbeing, regression analyses have shown that the feeling of mattering to others is significantly linked to overall life satisfaction and happiness [15]. These findings have been replicated, showing that mattering is not only strongly correlated with general life satisfaction, but also with social satisfaction and self-satisfaction [14]. This pattern persists when mattering and life satisfaction are assessed in specific contexts, such as a work environment. Mattering to others at work has been found to be strongly correlated with job satisfaction as well as with general life satisfaction [16].

It is clear that feeling that you matter has a positive impact on mental well-being, so the question arises whether feeling that you do not matter has the inverse effect. Flett [17] uses the term “anti-mattering” to describe this concept and states that not mattering to others is not simply the reverse of mattering; it is “qualitatively different” (p. 97). Flett [17] argues that while individuals who report high levels of mattering have had important psychological needs met, those who are high in anti-mattering not only have had their needs neglected, but rather violated, in such a way that the message they have received is that they are unworthy of attention and insignificant. Flett [17] developed the Anti-Mattering Scale (AMS) to assess anti-mattering and facilitate this line of research.

Feelings of not mattering to one’s mother, father, and friends are risk factors and are correlated with psychological distress. Even taking into account self-esteem, mastery, health conditions, and mental health history, feeling that one does not matter to others is predictive of depression [18,19]. Flett et al. [20] argued that it is imperative to address and foster mattering in university students as a protective factor for students’ mental health. Longitudinal studies have shown that those who feel that they do not matter to others in their lives are more vulnerable to depression over time [21].

### 1.4. Present Study

Very little research exists on the relationships between humor styles, loneliness, and feelings of not mattering. Given that affiliative and self-enhancing humor are both positively associated with self-esteem and negatively with depression, and that self-defeating humor is negatively associated with self-esteem and positively with depression, it is possible that feelings of not mattering will be found to be negatively correlated with the socially positive humor styles and positively correlated with self-defeating humor in the present study.

## 2. Method

### 2.1. Sample and Procedure

The data were collected from an online survey open to a university participant pool. The sample consisted of 254 undergraduate students (158 females, 96 males) enrolled in a first-year management study program at a Canadian university. The mean age was 18.73 years (*SD* = 1.99, range = 17 to 40). Participants were asked to indicate their age and gender, as well as respond to the measures outlined in the following section. Items from the three surveys were inter-mixed. The study received institutional ethics approval from NonMedical Research Ethics Board (NMREB), the University of Western Ontario (protocol approval number: 112755, date of approval: 11 October 2018).

### 2.2. Measures

#### 2.2.1. Humor Styles

The Humor Styles Questionnaire (HSQ; [3]) is a 32-item self-reported measure that assesses four humor styles (Cronbach’s alpha values as reported by Martin et al. [1]): affiliative (α = 0.80), self-enhancing (α = 0.81), self-defeating (α = 0.80), and aggressive humor (α = 0.77). Although the HSQ items are usually rated on a 7-point Likert scale (1 = totally disagree, 7 = totally agree), the present study used a 3-point Likert scale ranging from 1 = disagree to 3 = agree for consistency with the other measures and to explore the scale properties with this shorter response format.

#### 2.2.2. Loneliness

Three items from the revised UCLA Loneliness Scale [22] were used to assess participants’ feelings of loneliness. These three items have been used as a shortened version of the UCLA in many studies and have been shown to have good internal consistency, as well as convergent and divergent validity (α = 0.72; [23]). Specifically, participants were asked how often they felt that they lacked companionship, how often they felt left out, and how often they felt isolated from others. Items were rated on a 3-point scale from 1 = hardly ever to 3 = often. Responses are added for a total score; higher scores suggest greater loneliness.

#### 2.2.3. Anti-Mattering

The Anti-Mattering Scale (AMS; [17]) assesses individuals’ feelings of not mattering in their lives. This measure consists of five items measured on a 3-point scale from 1 = hardly ever to 3 = often. An example item on this scale is, “How often have you been treated in a way that makes you feel like you are insignificant?” A previous study has found good internal consistency for the AMS (α = 0.88; [24]).

## 3. Results

Because the AMS is a relatively new measure, the single factor structure was first assessed. A confirmatory factor analysis to test whether the AMS fit a single factor solution to the data was conducted using MPlus version 8 [25]. The results suggested that the single factor fit the data well in terms of fit indices: χ^2^(5) = 11.03, *p* = 0.051, CFI = 0.981, TLI = 0.962, RMSEA = 0.069, 90% CI [0.000, 0.125], SRMR = 0.025.

The internal consistency values for the loneliness and AMS were 0.82 and 0.79, respectively. The coefficient alpha values for the 3-point HSQ scales were compared to the values reported by Martin et al. [3] using the independent coefficient alpha test based on the chi-squared statistic created by Diedenhofen and Musch [26]. In the present study, affiliative humor had an alpha of 0.79, which was not significantly different to Martin et al.’s [3] value of 0.80 (χ^2^(1) = 0.094, *p* > 0.70). In contrast, the 3-point self-enhancing humor coefficient alpha was significantly lower (0.675 versus 0.81, χ^2^(1) = 24.70, *p* < 0.001). The 3-point aggressive humor also had a significantly lower alpha (0.643 versus 0.77, χ^2^(1) = 16.25, *p* < 0.001). In addition, self-defeating also had a significantly lower alpha for the 3-point response key (0.703 versus 0.80, χ^2^(1) = 13.29, *p* < 0.001). These results suggest that the original 7-point response key may result in more internally consistent estimates than the shorter 3-point scale, although the 3-point response key alpha values were still acceptable [27].

For each scale, average scale scores were generated. To assess for possible gender differences, an independent samples *t*-test was conducted. Age effects were assessed using correlations with the scale totals. Finally, all of the scale scores were correlated to determine how each construct was related to the others. Results of the independent samples *t*-test, correlations with age, and other descriptive statistics are in Table 1. The Levene’s *F*-test to compare group variances revealed that only the affiliative humor group variances were unequal, such that men were more variable in their responses than women were. As this *F*-test was significant, the pooled variance value was used for the affiliative humor *t*-test. Significant gender differences were found for the aggressive humor style and anti-mattering scores, such that men scored significantly higher on the aggressive humor style scale (moderate effect size) and women scored significantly higher on the anti-mattering scale (small to moderate effect size). No other significant gender differences were found. Age did not correlate significantly with the scale scores.

The descriptive statistics for the complete sample and inter-scale correlations are in Table 2. Loneliness has a robust positive correlation with anti-mattering. The two positive humor styles (affiliative and self-enhancing humor) negatively correlated with both loneliness and anti-mattering. Self-defeating humor positively correlated with loneliness and anti-mattering. Aggressive humor did not correlate significantly with either loneliness or anti-mattering.

## 4. Discussion

From the current study, it is clear that self-reported loneliness is highly related to anti-mattering, not only from the direct correlation between them, but also the similarity of the magnitude and direction of the correlations with both constructs with each of the humor styles. The correlation between the loneliness and anti-mattering dimensions in this study replicated the value reported by Flett et al. [2]. To further explore the relationship between anti-mattering and loneliness, the correlations with self-reported humor styles were examined. No distinctions could be made based on the correlations as both anti-mattering and loneliness correlated positively with self-defeating humor, negatively with affiliative and self-enhancing humor, and did not correlate significantly with aggressive humor.

If loneliness and anti-mattering are so closely related, where does anti-mattering fit within theories of loneliness? Loneliness research has described affective and cognitive manifestations, as well as antecedents of possible childhood neglect and number of social relationships [28,29]. The focus is usually on changes in social relationships and many articles refer to the impact of “quality” in relationships [30], but few discuss how the quality of the relationship alleviates feelings of loneliness. In fact, the definitions usually state that loneliness is one’s own dissatisfaction with relationships, putting the onus on the lonely individual. Results such as the present study and from Flett and colleagues [2] expand the definition, suggesting that it may not just be one’s dissatisfaction with relationships that creates loneliness, but rather the actions of others have evoked the feeling that one does not matter. Perhaps, at its core, loneliness and the associated negative outcomes are not completely due to the absence of a satisfying social network, but due to the presence of a deep belief of insignificance.

Women in the present study were more likely than men to report feeling that they do not matter. Interestingly, Taylor and Turner [21] found that women were more likely to report that they do matter to others, but also identified sex differences emerged in the experience of mattering. Notably, for women, mattering is highly correlated with mental health, but for men, mattering is positively related to the experience of mastery and social support [21]. Flett [17] has argued that anti-mattering is not simply the opposite of mattering and has found a trend across studies that women score higher on anti-mattering (Flett et al., unpublished manuscript).

Men were more likely than women to use aggressive humor, which is consistent with previous research. According to Martin et al. [3], aggressive humor is negatively associated with femininity and positively associated with masculinity. Dyck and Holtzman [5] found trends that suggested aggressive humor in men is associated with increased perceived social support, but for women, higher ratings of aggressive humor is associated with decreased perceived social support. Sillars et al. [31] also found that men were more likely to use aggressive humor and suggested that this is due to continued gender norms of men using joking to push boundaries while adhering to social norms. Weisfeld [32] likened aggressive humor to play-fighting, which, from an evolutionary standpoint, is seen as practice and valuable for evolutionary fitness. Men may be more likely to use aggressive humor because it presents evolutionary and social benefits.

The current study also assessed psychometric properties of two measures. A confirmatory factor analysis found that the AMS is a unidimensional scale, which was the intention of its author. For the HSQ, the focus was on whether the response format could be changed from a 7-point to a 3-point scale. Results indicated that this change significantly reduces the internal consistency estimates for three of the four scales. It is therefore suggested that future work with the HSQ maintains a longer response format, although the 3-point format still resulted in acceptable coefficient alpha values.

### 4.1. Limitations and Future Directions

One limitation of the present study is that the sample were undergraduate students. How these results generalize to the general population requires further research. Another potential limitation is that only self-reported measures were used to assess humor styles, loneliness, and feelings of not mattering. As with all self-reported measures, there is a possibility of social desirability bias influencing participants’ responses. Participants may have underreported their feelings of loneliness and anti-mattering in order to present themselves in a more favourable light (although mean scale scores were greater than 1.5 out of a maximum score of 3). Research may want to investigate peer reports [33] or behavioral indicators of loneliness [34] and anti-mattering in future studies.

Future research may also want to assess the relationships between humor styles, loneliness, and anti-mattering using different measures. For example, although the three item loneliness scale has been found to be effective in other studies [6], and the UCLA Loneliness Scale [22] is a frequently used measure in the loneliness literature, Lasgaard et al. [35] report that different aspects of loneliness (peer-related versus family-related) may have different effects on psychological expressions. What is unknown is how humor styles and anti-mattering correlate with loneliness when certain types of loneliness are assessed, as the present results can only conclude that anti-mattering and overall loneliness are highly correlated.

Future research may also want to examine the joint relationships between humor styles, loneliness, and anti-mattering with respect to the possibly superordinate construct of depression using behavior genetic analyses. For example, loneliness has been conceptualized by some researchers to be a component of depression [36]. As loneliness has been found to have a genetic component, with heritability estimates around 35% [37], and because loneliness demonstrates significant genetic correlations with humor styles [6], suggesting common genetic factors influence the co-occurrence of loneliness and humor styles, these common factors may be from generalized depression. Recently Kfrerer et al. [38] reported that there were both significant phenotypic (observed) and genetic correlations between the humor styles of affiliative (negative direction), self-enhancing (negative direction), and self-defeating (positive direction) with a depressed affect. How anti-mattering fits within the constructs of loneliness, humor styles, and depression at genetic and environmental levels is a research topic that may help to clarify if loneliness and anti-mattering are facets of depression.

### 4.2. Implications and Conclusions

This study demonstrated the important role of humor styles as psychosocial construct. The pattern of association with loneliness and mattering suggests that one’s humor styles may be a behavioral indicator of psychosocial functioning, but also an avenue to psychological health. The present study provides further evidence that anti-mattering and loneliness possess strikingly similar qualities and may in fact be one concept. In clinical intervention for loneliness, these findings implore treatments that focus on relationship development and involve others, such as family therapy.

## Figures and Tables

**Table 1:** Descriptive statistics and independent samples *t*-tests.

Measures	Men Average (SD)	Women Average (SD)	Levene’s *F*	*t*	Cohen’s *d*	Correlation with Age
Loneliness	1.71 (0.57)	1.83 (0.54)	1.39	−1.64	−0.21	0.09
Anti-mattering	1.68 (0.46)	1.82 (0.44)	1.97	−2.32 *	−0.30	0.10
Affiliative humor	2.52 (0.46)	2.53 (3.00)	5.81 *	−0.18	−0.02	−0.10
Self-enhancing humor	2.14 (0.35)	2.09 (0.39)	2.55	1.01	−0.13	−0.06
Aggressive humor	1.82 (0.36)	1.62 (0.35)	0.01	4.05 *	0.52	−0.07
Self-defeating humor	1.77 (0.38)	1.80 (0.41)	0.16	−0.57	−0.07	0.01

* *p* < 0.05, two-tailed.

**Table 2:** Descriptive statistics and inter-scale correlations.

Measure	Mean	SD	1	2	3	4	5	6
1. Loneliness	1.78	0.55						
2. Anti-mattering	1.77	0.45	0.65 **					
3. Affiliative humor	2.52	0.41	−0.33 **	−0.22 **				
4. Self-enhancing humor	2.10	0.37	−0.25 **	−0.19 **	0.24 **			
5. Aggressive humor	1.70	0.36	−0.05	−0.04	−0.05	0.11		
6. Self-defeating humor	1.79	0.39	0.21 **	0.25 **	−0.04	0.11	0.21 **	

* p < 0.05; ** *p* < 0.01; two-tailed.
